# The rise of 3D spheroids in radiobiology for assessing tumour radioresistance

**DOI:** 10.2340/1651-226X.2026.45079

**Published:** 2026-02-03

**Authors:** Alexandra Charalampopoulou, Fabrizio De Luca, Giuseppe Magro, Niloufar Matoor, Amelia Barcellini, Giorgio Butella, Giovanni Battista Ivaldi, Sara Lillo, Lorenzo Manti, Alessio Mereghetti, Paola Tabarelli De Fatis, Maria Grazia Bottone, Angelica Facoetti

**Affiliations:** a Radiobiology Unit, Research and Development Department, CNAO National Center for Oncological Hadrontherapy, Pavia, Italy; b Hadron Academy PhD Course, School for Advanced Studies (IUSS), Pavia, Italy; c Department of Biology and Biotechnology L. Spallanzani, University of Pavia, Pavia, Italy; d Medical Physics Unit, Clinical Department, CNAO National Center for Oncological Hadrontherapy, Pavia, Italy; e Radiation Oncology Unit, Clinical Department, CNAO National Center for Oncological Hadrontherapy, Radiation Oncology Unit, Clinical Department, Pavia, Italy; f Department of Internal Medicine and Therapeutics, University of Pavia, Pavia, Italy; g Research and Development Department, CNAO National Center for Oncological Hadrontherapy, Pavia, Italy; h Radiation Oncology Department, Istituti Clinici Scientific Maugeri IRCCS, Pavia, Italy; i Department of Mathematics and Physics, University of Campania “L. Vanvitelli”, Caserta, Italy; j National Institute for Nuclear Physics (INFN), Naples Section, Naples, Italy; k Medical Physics Unit, Clinical Scientific Institutes Maugeri IRCCS, Pavia, Italy

**Keywords:** Particle therapy, conventional radiotherapy, glioblastoma, osteosarcoma, 3D tumour spheroids, relative biological effectiveness (RBE), radioresistance

## Abstract

**Background and purpose:**

Particle therapy (PT), including proton (PRT) and carbon ion radiotherapy (CIRT), offers physical and biological advantages over photon radiotherapy (XRT), particularly for radioresistant tumours such as glioblastoma and osteosarcoma. However, systematic preclinical comparisons using physiologically relevant models remain limited.

**Material and methods:**

T98G (glioblastoma), Saos-2 and U2-OS (osteosarcoma) cells were cultured as two-dimensional (2D) monolayers and three-dimensional (3D) spheroids and irradiated with XRT, PRT or CIRT at 2, 4 or 6 Gy. Clonogenic survival, metabolic activity (PrestoBlue), invasion and spheroid growth kinetics were quantified. Relative biological effectiveness (RBE) was derived from survival data, and spheroid sections were analysed histologically (H&E).

**Results:**

CIRT induced the strongest cytotoxic and anti-invasive effects across all models. In 2D cultures, the surviving fraction at 2 Gy decreased from 0.62 to 0.69 after XRT to 0.16–0.28 following CIRT (RBE = 2.1–2.4 vs. 1.1 for PRT; *p* < 0.0001). 3D spheroids exhibited overall higher radioresistance, yet CIRT markedly reduced growth and invasion, lowering normalised indices to 0.52 ± 0.12 (T98G) and 0.58 ± 0.09 (Saos-2) at 6 Gy, while photons often promoted invasion (> 1.2; *p* < 0.001). RBE values in 3D reached 3.6–4.0. H&E staining confirmed dose-dependent architectural disruption, with carbon ions inducing extensive necrosis and cellular degeneration.

**Interpretation:**

This study introduces a robust 3D preclinical platform for radiobiological assessment of particle therapy. CIRT consistently overcame intrinsic and microenvironment-mediated resistance, outperforming photons and protons in suppressing viability, invasion and spheroid integrity, thus reinforcing the translational relevance of 3D models and the therapeutic promise of carbon ion therapy for resistant malignancies.

## Introduction

One major limitation of radiation therapy (RT) is the presence of tumours with intrinsic radioresistance, often driven by biological factors. Despite distinct origins, glioblastoma and osteosarcoma both exhibit marked resistance to conventional RT (CRT), highlighting the role of tumour biology in treatment outcomes [1]. Glioblastoma, likely arising from astrocytes or neural precursors, is highly aggressive and radioresistant [2], while osteosarcoma, from osteoblasts, poses similar challenges [3]. Their CRT resistance makes both promising candidates for particle RT (PT), including proton therapy (PRT) and carbon ion therapy (CIRT), which offer improved dose conformity and tissue sparing [4–6]. Carbon ions are high-LET, inducing dense DNA damage, whereas protons have relatively low-linear energy transfer (LET), rising near the distal Bragg peak, enhancing effectiveness in radioresistant and hypoxic tumours [7]. Despite these advantages, PT remains controversial, with limited clinical evidence and challenges in conducting phase III trials for rare or difficult-to-treat tumours [8–10]. Advanced experimental models are thus essential to validate PT’s biological effects and optimise clinical use. Beyond clonogenic survival, radiation quality can differentially modulate tumour cell migration and invasion. While high-LET radiation such as carbon ions generally suppresses invasive behaviour through severe and irreversible cellular damage, low-LET irradiation, including photons and protons, has been reported to induce transient, stress-mediated pro-migratory responses in surviving cells [11].

Two-dimensional (2D) cell cultures have long been used to study cellular radiation responses [12–14], with clonogenic assays considered the ‘gold standard’ for assessing survival and calculating relative biological effectiveness (RBE) [15]. However, 2D systems oversimplify *in vivo* conditions, altering cell–cell and cell–matrix interactions crucial for proliferation, metabolism, differentiation and function [16, 17]. Recent advances in three-dimensional (3D) culture technologies, particularly tumour spheroids, more accurately replicate tissue architecture and microenvironmental features [18, 19]. Spheroids, easily cultured in standard laboratory settings, provide a practical and physiologically relevant alternative to 2D models, with marked effects on proliferation, survival and radiation response. In this study, we evaluated and compared 2D and 3D cell cultures of human glioblastoma and osteosarcoma exposed to photons, protons and carbon ions, assessing long-term survival, short-term viability, invasion and spheroid growth. To the best of our knowledge, this is the first work directly comparing these parameters and RBE across dimensional models and multiple radiation types.

## Material and methods

The T98G (human glioblastoma multiforme) cell line was purchased from Sigma-Aldrich (St. Louis, MO, USA), while the Saos-2 and U2-OS (human osteosarcoma) cell lines were kindly provided through the collaboration with the BIOHOT project, funded by the National Institute for Nuclear Physics (INFN). T98G cells were grown in Eagle’s Minimum Essential Medium with the addition of 10% heat-inactivated foetal bovine serum (FBS), 100 U/mL penicillin and 0.1 mg/mL streptomycin. Saos-2 cells were maintained in McCoy’s 5A (Modified) Medium containing 15% heat-inactivated FBS, 100 U/mL penicillin and 0.1 mg/mL streptomycin and U2-OS in McCoy’s 5A (Modified) Medium with 10% heat-inactivated FBS, 100 U/mL penicillin and 0.1 mg/mL streptomycin. All cell lines were cultured in a humidified atmosphere at 37°C containing 5% CO_2_. Culture media and all supplements were obtained from Sigma Aldrich. Media were changed every 2–3 days, and cells were subcultured using 0.25% trypsin when approximately 80% confluent.

### Clonogenic survival assay

Sham-irradiated (control) and irradiated cells were counted using the LUNA-II automated cell counter (Logos Biosystems, Villeneuve d’Ascq, France), and the desired number for each dose and type of irradiation was seeded into triplicates and incubated for 10 days in the case of T98G and U2-OS cells and 14 days for Saos-2 cell line. Seeding numbers for each irradiation condition are reported in Table 1. Following incubation, the media were removed, and colonies were fixed with 70% cold ethanol, stained with 0.1% of crystal violet solution, and let air-dry overnight. Afterwards, colonies were counted, keeping a cut-off of at least 50 cells per cluster. The average of the three colony counts was divided by the number of cells plated, giving the plating efficiency (PE). The surviving fractions (SF) were determined by dividing the PE of the treated cells by the PE of the controls and then multiplying by 100. SFs were plotted on a semilog scale as a function of the dose and survival data were fitted using the linear quadratic (LQ) model obtaining the α, β and the ratio of α/β parameters. Radioresistance was assessed by calculating the RBE, defined as the ratio of the absorbed dose of the reference radiation to that of the radiation under investigation at the D10 level, where D10 represents the dose required to reduce cell survival to 10%.

**Table 1:** Cell numbers seeded per dose for clonogenic survival assays across all irradiation types for U2-OS, Saos-2 and T98G cell lines.

Cell line	0 Gy	0.5 Gy	1 Gy	2 Gy	4 Gy	6 Gy
XRT						
U2-OS	500	600	600	900	5000	11,000
Saos-2	500	750	600	1000	5500	12,000
T98G	250	300	350	650	900	1250
PRT						
U2-OS	500	600	600	900	5000	11,000
Saos-2	500	750	600	1000	5500	12,000
T98G	250	300	350	650	900	1250
CIRT						
U2-OS	500	1200	1200	1800	8000	17,000
Saos-2	500	1500	1600	2000	9000	20,000
T98G	250	600	700	1300	1800	2500

Studies demonstrated a correlation between the surviving fraction at 2 Gy (SF2) in cancer cell lines and the clinical radioresponsiveness of the corresponding tumour types [20]. Accordingly, in our study, we also assessed the intrinsic radiosensitivity of the selected cell lines by evaluating their SF2 values following exposure to different radiation modalities, a widely recognised biological marker for predicting tumour response to RT. Three independent experiments were performed for each condition.

### MTT assay

The impact of different irradiation types on the viability of each cell line was evaluated using the MTT assay. Both sham-irradiated and irradiated cells were plated at a density of 1–3 × 10^4^ cells per well in 96-well plates, with a total volume of 100 μL per well and incubated at 37°C in a humidified atmosphere and 5% CO₂. At 24 h and 5 days post-seeding, 10 μL of MTT solution (Sigma Aldrich) was added to each well, followed by incubation for 4 h at 37°C in the dark. Subsequently, the resulting formazan crystals were solubilised in 100 μL of DMSO. Absorbance was measured at 570 nm using a BioTek® 800TM TS microplate reader equipped with Gen5TM software. Cell viability was expressed as a percentage, calculated by the ratio of the average optical density (OD₅₇₀) of treated samples to that of control samples, multiplied by 100, as follows:

Percentage of cell viability = (Average OD₅₇₀-treated cells / Average OD₅₇₀ control cells) × 100 [21, 22].

### Cell invasion assay

Cell invasive capacity was assessed using the BioCoat^TM^ Matrigel® Invasion assay (Corning, MA, USA), which consists of 8.0 µm pore-size PET membrane inserts pre-coated with a reconstituted basement membrane matrix (Matrigel). In the BioCoat^TM^ Matrigel® invasion system used in this study, the inserts are pre-coated with growth factor–reduced Matrigel, allowing invasion to be primarily driven by the chemoattractant gradient rather than by matrix-derived growth factor signalling. Following irradiation, cells were detached and counted. Depending on the cell line, radiation modality and dose, between 1.0 × 10⁵ and 2.0 × 10⁵ cells were seeded into the upper chamber of each insert. The lower wells were filled with complete growth medium containing 20–30% FBS to serve as a chemoattractant, facilitating directional invasion through the Matrigel-coated membrane. After 24 h of incubation at 37°C in a humidified 5% CO₂ atmosphere, non-invading cells and residual Matrigel were gently removed from the upper surface of the membrane using a sterile cotton swab. Invaded cells adhering to the underside of the membrane were fixed in 70% cold ethanol and stained with crystal violet. Invasive cells were visualised using a phase-contrast microscope (Olympus IX71) at 20X magnification, and five randomly selected fields per insert were imaged for quantification.

Data were expressed as the percentage of invasion relative to the non-irradiated control group (set as 100%) and presented as mean ± standard deviation (SD). Each experimental condition was repeated in at least three independent biological replicates [23, 24].

### 3D spheroids growth

Spheroid formation was achieved by using U-bottom 96-well culture plates (Corning, Costar, NY, USA), with one spheroid formed per well, using SpheroTribe solution according to the manufacturer’s instructions (Idylle, France) [25]. For each cell line, optimal seeding densities were selected based on preliminary experiments aimed at ensuring long-term spheroid viability, as described below. Cells were seeded and cultured in a humidified atmosphere at 37^°^C containing 5% CO_2_ for 72 h in order to form aggregates in a total volume of 100 μL, enough to fill each well. Afterwards, spheroids were treated with single doses of different types of RT. The medium was refreshed every 3 days, while spheroid growth was assessed by capturing images with a phase-contrast microscope (Olympus IX71) before irradiation and every 24 h up to 5 days post-irradiation. The diameter was then measured using ImageJ v1.54p software (NIH, Bethesda, MA, USA).

### 3D spheroids viability

To monitor spheroid health over time and in order to decide the optimal cell density per spheroid for each cell line, the T98G, Saos-2 and U2-OS cancer cell lines were seeded at increasing densities and cultured for 10 days. The condition of cells from spheroids at the seeding densities of 5 × 10^4^, 7.0 × 10^4^, 1.0 × 10^4^ and 1.5 × 10^4^ cells/well was assessed using the PrestoBlue cell viability reagent (ThermoFisher Scientific, USA), which detects the reducing power of live cells and was evaluated over the period of 10 days. More specifically, PrestoBlue Cell Viability Reagent was diluted 1:10 into the medium of each well of 96-well plates, which were then incubated at 37°C and 5% CO_2_ for 10 min before reading on an absorbance microplate reader at 570 nm, using 600 nm as a reference wavelength (normalised to the 600-nm value). The absorbance values were normalised by spheroid size; higher ratios indicate healthier spheroids. This assay was also employed to assess the viability of both irradiated and non-irradiated spheroids every 24 h over a 5-day period.

For each experiment, at least 16 representative spheroids [26] per irradiation dose were chosen for every cell line, and each experiment was conducted three times.

### 3D relative biological effectiveness

To assess the impact of irradiation on spheroid growth, we calculated the growth rate of spheroids from the changes in their diameters over time. The initial diameter of each spheroid was measured prior to irradiation, and subsequent measurements were taken at regular intervals (e.g. 24, 48, 72, 96 and 120 h post-irradiation). For spheroid size quantification, 16 independent spheroids per condition per experiment were analysed, derived from at least three independent experiments. Measurements were performed on the same spheroids throughout the observation period to enable longitudinal assessment of growth dynamics.

The growth rate for each spheroid was calculated using the following formula:


$$Growth\;rate=\frac{Final\;diameter\;-Initial\;diameter}{Time\;inervel}$$

For each condition, the average growth rate was calculated based on the measurements of multiple spheroids.

To quantify the biological effect of the different types of irradiation, we determined the growth inhibition for each irradiated group relative to the control group (mock-treated spheroids). This was calculated using the formula:


$$Growth\;Inhibition=1-\frac{Growth\;Rate\;of\;Irradiated\;Group}{Growth\;Rate\;of\;Control\;Group}$$

The 3D-RBE was calculated by comparing the growth-inhibitory biological effect of the test irradiation to the reference radiation (Photon RT) at the same level of biological effect.

### 3D spheroids invasion

Matrigel® Basement Membrane Matrix (Corning) was thawed overnight at 4°C on ice and diluted to a final concentration of 5 mg/mL using ice-cold, freshly prepared complete growth medium. To preserve the integrity of the matrix and prevent premature solidification, all materials and reagents in contact with Matrigel were maintained on ice throughout the procedure. Using pre-chilled pipette tips, 50 μL of the diluted Matrigel solution was carefully dispensed into each well of a pre-cooled, low-adhesion 96-well plate and evenly distributed. The plate was then incubated at 37°C for 30 min to allow the Matrigel to polymerise and form a stable three-dimensional scaffold.

Following matrix solidification, individual pre-formed spheroids were gently implanted into the centre of each well. Invasive outgrowth was monitored using phase-contrast microscopy (40X magnification), with images captured immediately after implantation (baseline) and at 24-h intervals for up to 5 days. Digital images were acquired without post-processing to maintain the integrity of the raw data [24].

### Irradiations

T98G, Saos-2 and U2-OS cells, cultured in T25 or T75 culture flasks (Falcon and Corning, respectively), were irradiated at the phase of their exponential growth with 2, 4 or 6 Gy of either photon or proton or carbon ion beams. Spheroids from the same cell lines were cultured in U-bottom 96-well culture plates and irradiated 72 h after seeding. Samples that were not exposed to irradiation were treated similarly to the irradiated ones to ensure identical experimental conditions, with the exception of radiation exposure. During irradiation procedures, control samples were kept outside the irradiation room on a laboratory bench at room temperature for the same duration as the corresponding irradiation and were returned simultaneously with irradiated samples to standard cell culture conditions (37°C, 5% CO₂).

Photon RT was performed in the Radiotherapy Department of the Istituti Clinici Scientifici Maugeri of Pavia (Italy), using a 6 MV beam from a LINAC linear accelerator. Flasks or culture plates were horizontally positioned above a 1.5-cm thick layer of Plexiglas, enough to ensure electronic equilibrium. Samples were irradiated from below (180°) with 1, 2, 3, 4, 5 and 6 Gy.

Proton and carbon ion irradiations were performed with the fixed clinical beam lines using the active scanning technique at the National Center for Oncological Hadrontherapy (CNAO) of Pavia, Italy. T12.5 flasks were filled to the neck with non-complete medium and positioned into a water phantom with its entrance window located at the room isocentre, while 96-well culture plates were horizontally positioned on a robotic couch and irradiated using the vertical beamline.

A spread-out Bragg peak (SOBP) was created to deliver a homogenous physical dose across the target volume by modulating 16 beam energies (130.6–163.6 MeV/u) in the case of protons and 31 beam energies (246–312 MeV/u) for carbon ions. More specifically, T12.5 flasks were centred in the SOBP with cells placed at a depth of 150 mm corresponding to the middle of the SOBP, while 96-well culture plates were placed below 143 mm of solid water (RW3, plastic). Samples were exposed to the same doses as those used with photon RT.

Irradiations were delivered at clinically relevant dose rates. Photon irradiation was performed at a dose rate of 3 Gy/min, while proton and carbon ion irradiations were delivered at average dose rates of 1.26 Gy/min and 0.632 Gy/min, respectively. Consequently, irradiation times varied according to radiation modality and prescribed dose, with particle irradiations requiring longer delivery times than photon irradiation.

Immediately after both types of irradiation, non-complete media were aspirated from the flasks and replaced with complete medium.

Although samples were irradiated across a dose range of 1–6 Gy, subsequent functional analyses were conducted primarily at 2 Gy, corresponding to a CRT fraction, and at 4 and 6 Gy, representing higher per-fraction doses commonly used in hypofractionated or dose-escalation studies.

### 3D spheroids histology

Haematoxylin-eosin (H&E) staining was performed on sections of spheroids that had been cultured for 7 days following irradiation exposure to assess their morphological alterations. The spheroids were fixed in a 4% formalin neutral buffer solution (Sigma Aldrich, Milan, Italy). Subsequently, samples were kept in absolute ethanol, followed by acetone and finally embedded in Paraplast X-TRA (Sigma Aldrich, Milan, Italy). Serial sectioning of the spheroids was carefully carried out using a manual rotary microtome. Sections 6 microns thick were cut and collected on silane-coated slides.

H&E staining was performed as previously reported [27]. Briefly, serial spheroid sections were processed as follows: staining with 1% eosin solution (Bio-Optica Milano S.p.A., Milano, Italy), counterstaining with Carazzi’s Haematoxylin (Bio-Optica Milano S.p.A., Milano, Italy), followed by 20 min wash in running tap water; the proteins stain pink to red, while the nucleic acids stain dark blue to violet. Lastly, all stained sections were dehydrated in ethanol, cleared in xylene and mounted in Eukitt (Kindler, Freiburg, Germany). Approximately 10 randomised sections (5 microscopic fields) per experimental condition were examined by a blinded operator using a Leica DM6B WF bright-field microscope (Leica Microsystems, Buccinasco, MI, Italy), and the images acquired with a Leica dfc 7000 t CCD camera.

### Statistical analysis

Clonogenic survival curves were generated using SDAnext (v0.0), an open-source software for survival data analysis developed by Magro, G. (2025), available on GitHub (https://github.com/BeppeMagro/SDAnext) and licensed under the Apache License 2.0. Statistical analysis included linear regression for dose–response relationships, two-way ANOVA for main effects and interactions and post hoc comparisons using Tukey’s or Bonferroni’s multiple comparison tests, as appropriate. A *p*-value ≤ 0.05 was considered statistically significant. All statistical analyses were conducted using R version 4.2.2.

## Results

### 2D cell cultures

After the exposure to different doses of photons, protons or carbon ions, the radioresistance of all cell lines was first determined in a 2D environment, by means of clonogenic survival. PE of control non-irradiated T98G, U2-OS and Saos-2 cells was 37%, 35% and 34%, respectively.

**Figure 1:**
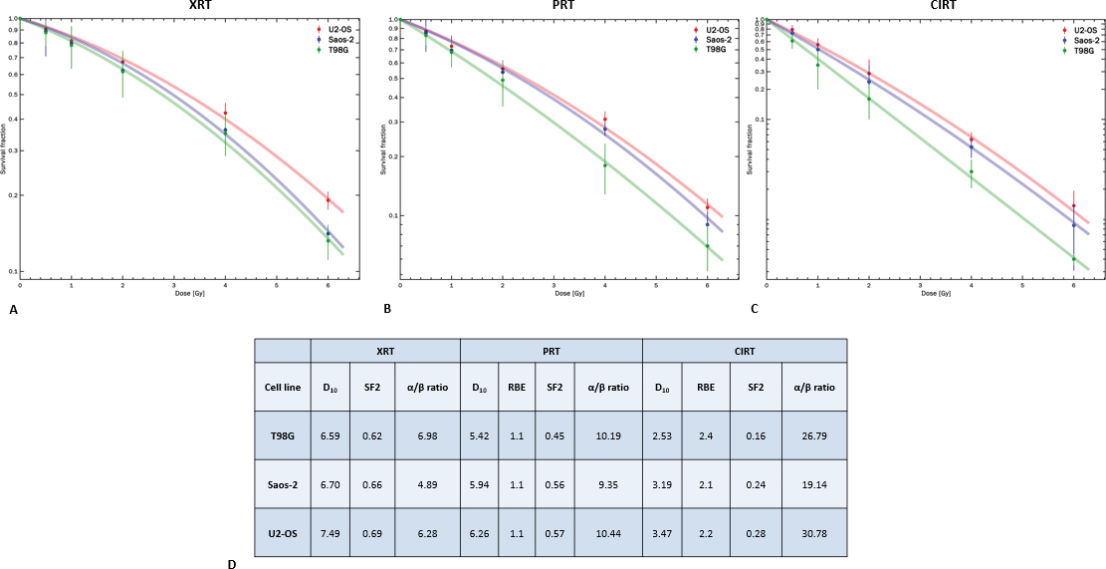
Survival curves of T98G, U2-OS and Saos-2 cell lines after the exposure to (A) photons, (B) protons and (C) carbon ions. Distinct scales were used for each graph to more accurately represent the differences between cell lines and irradiation conditions. (D) Corresponding D_10_, SF2, α/β ratio and RBE values corresponding to photons, protons and carbon ions across the three different cell lines.

The comparison of colony numbers between irradiated and untreated control samples indicated that all cell lines were markedly more sensitive to carbon ions than to photons or protons at the same physical dose (Figure 1). Following photon irradiation, U2-OS cells exhibited the highest radioresistance, with a D₁₀ of 7.49 Gy and an α/β ratio of 6.28, followed by Saos-2 cells (D₁₀ = 6.70 Gy; α/β = 4.89). T98G cells, although showing the highest α/β ratio of 6.98, had an SF2 value of 0.62, indicating intermediate radiosensitivity (Table 1). Proton irradiation produced a similar pattern, with survival responses largely consistent with photon exposure; U2-OS remained the most radioresistant, followed by T98G and Saos-2, with α/β ratios of 10.44, 10.19 and 9.35, respectively (Figure 1B). Upon carbon ion exposure, all cell lines demonstrated enhanced radiosensitivity, yet U2-OS cells retained the highest resistance, followed by Saos-2, while T98G cells were the most sensitive. This trend was reflected in the RBE values, highlighting the effectiveness of carbon ions in overcoming the relative radioresistance observed with photons and protons (Figure 1C).

The RBE of all cell lines was found to be 1.1 for protons when compared to photons, in line with its clinically adopted value. In contrast, the RBE values for carbon ions relative to photons varied across the different cell lines. Specifically, Saos-2 cells exhibited an RBE of 2.1, T98G cells had an RBE of 2.4 and U2-OS cells demonstrated an RBE of 2.2, highlighting the differential biological response of the cell lines to carbon ions relative to photons.

Across all irradiation modalities, U2-OS cells consistently exhibited the highest SF2 values, indicative of higher intrinsic radioresistance, followed by Saos-2, while T98G cells showed the lowest SF2 values, reflecting greater susceptibility to radiation-induced cell death. More specifically, SF2 values for T98G were 0.62 following photon irradiation, 0.45 after proton irradiation and 0.16 after carbon ion irradiation. For U2-OS cells, the corresponding SF2 values were 0.69, 0.57 and 0.28, while Saos-2 cells exhibited SF2 values of 0.66, 0.56 and 0.24 (Figure 1D).

**Figure 2:**
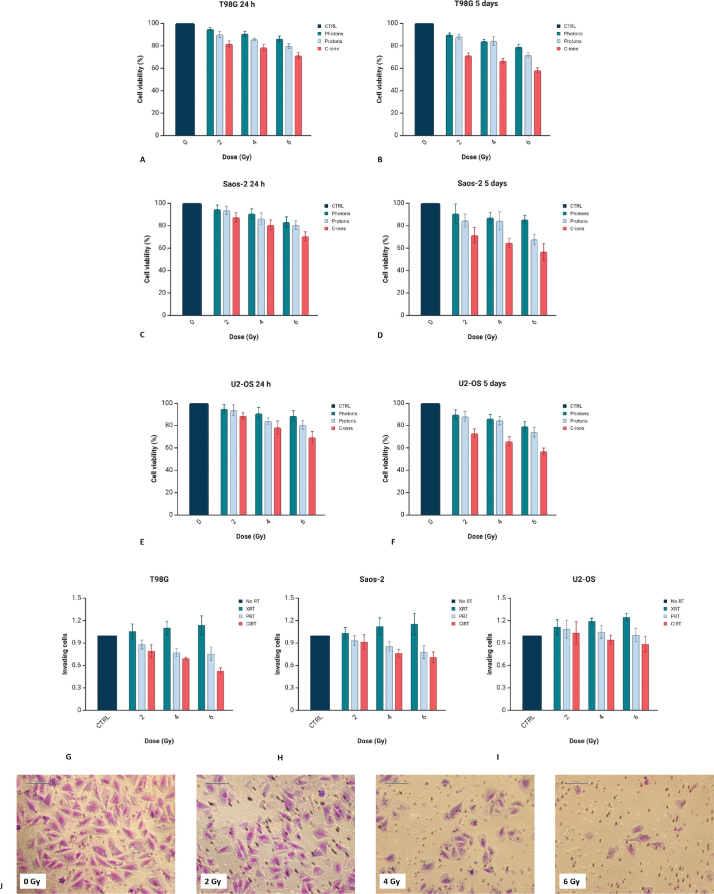
Cell viability of T98G (A, B), Saos-2 (C, D) and U2-OS (E, F) cell lines evaluated by MTT assay at 24 h and 5 days following irradiation with photons, protons and carbon ions at doses of 2, 4 and 6 Gy. Viability is reported as a percentage relative to non-irradiated controls and presented as mean ± standard deviation. Invasion of (G) T98G, (H) Saos-2 and (I) U2-OS cells after exposure to XRT, PRT or CIRT. Graphs created with BioRender.com. (J) Representative images showing invasion of U2-OS cells under control conditions and following carbon ion irradiation at 2, 4 and 6 Gy, acquired at 20X magnification.

MTT assay analyses showed that cell viability remained largely unchanged across all cell lines at 24 h post-irradiation, irrespective of radiation modality or dose (Figure 2A, C, E). By 5 days post-irradiation, however, a significant, dose-dependent decrease in viability was observed. Carbon ions consistently induced the greatest reduction in cell viability compared to protons and conventional X-rays in all cell lines (Figure 2). For instance, in T98G cells, viability at 5 days decreased to 90.6% ± 4.3% at 2 Gy and 80.0% ± 5.2% at 6 Gy after photons, compared to 71.4% ± 4.2% and 56.6% ± 4.1% after carbon ions. Similarly, U2-OS cells showed 89.8% ± 2.4% at 2 Gy and 72.9% ± 2.1% at 6 Gy with photons, versus 75.0% ± 2.4% and 55.4% ± 2.1% with carbon ions. Overall, photons and protons induced modest decreases in viability, whereas carbon ions caused a pronounced reduction at both conventional (2 Gy) and higher doses. Two-way ANOVA confirmed significant main effects of dose and radiation type (*p* < 0.0001), with no significant interaction (*p* = 0.686).

Regarding invasion, T98G cells showed a dose-dependent increase following exposure to photons, with relative values of 1.06 ± 0.10 at 2 Gy (*p* = 0.01552 vs. CIRT), 1.10 ± 0.09 at 4 Gy (*p* = 0.002087 vs. PRT) and 1.14 ± 0.12 at 6 Gy (*p* = 0.0003 vs. PRT). In contrast, protons significantly reduced invasion of 0.88 ± 0.06, 0.77 ± 0.05 and 0.76 ± 0.09 (*p* = 0.04805 vs. CIRT), respectively, while carbon ions led to the strongest suppression of 0.79 ± 0.09, 0.69 ± 0.01 and 0.53 ± 0.04 (Figure 2G). In Saos-2 cells, photons increased invasion to 1.03 ± 0.07, 1.12 ± 0.11 and 1.16 ± 0.14 (*p* = 0.001588 vs. PRT), proton irradiation moderately reduced it (0.93 ± 0.07, 0.86 ± 0.06 and 0.78 ± 0.08), and exposure to carbon ions had the most pronounced inhibitory effect reducing invasion to 0.91 ± 0.09, 0.76 ± 0.05 and 0.71 ± 0.06 (Figure 2H). U2-OS cells showed a similar trend, with XRT promoting invasion in a dose-dependent manner (1.11 ± 0.10 at 2 Gy, 1.19 ± 0.04 at 4 Gy and 1.25 ± 0.05 at 6 Gy), while PRT had more variable effects (1.09 ± 0.12, 1.05 ± 0.09 and 1.01 ± 0.09). CIRT reduced invasion mostly at higher doses of 4 and 6 Gy (0.94 ± 0.06 and 0.89 ± 0.10) (Figures 2I, J).

### 3D cell cultures

**Figure 3:**
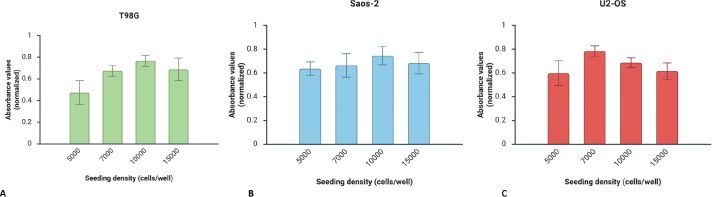
Spheroid cell health assessment using PrestoBlue cell viability reagent for (A) T98G, (B) Saos-2 and (C) U2-OS cells. Graphs created with BioRender.com.

Firstly, the evaluation of cell health of T98G, U2-OS and Saos-2 cells using the PrestoBlue cell viability assay showed that all spheroids were viable and healthy during 10 days of incubation. The optimal cell density per spheroid was different for each cell line, corresponding to 1.0 × 10^4^ cells/spheroid for T98G and Saos-2 cells and 7.0 × 10^4^ cells/spheroid for U2-OS cell line (Figure 3). The seeding densities shown in Figure 3 represent independent optimisation conditions and are reported for descriptive purposes.

Afterwards, the effects of the different types of irradiation were evaluated using the 3D spheroids generated from each cell line, in terms of growth rate. As seen in Figure 4, control non-irradiated spheroids of all cell lines continued to grow in terms of diameter length, presenting an increment of almost 30 ± 9% for T98G cells, 28% ± 6% for Saos-2 and 46% ± 8% for U2-OS with these values representing the growth at the final time point relative to the initial measurement taken at the first time point.

**Figure 4a:**
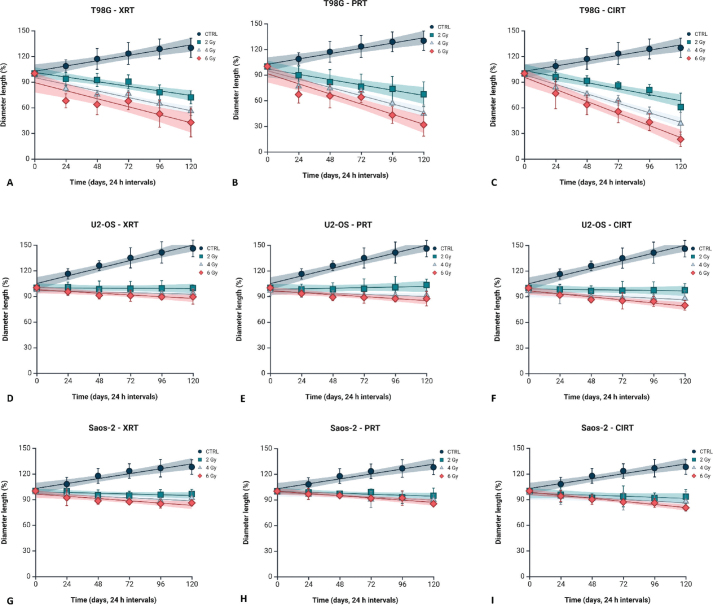
Growth curves of 3D spheroids derived from T98G (A–C), Saos-2 (D–F) and U2-OS (G–I) cell lines, comparing non-irradiated controls to spheroids exposed to photon, proton and carbon ion irradiation. Baseline measurements (time point 0) correspond to spheroid size before irradiation. Subsequent measurements were performed at 24-h intervals, spanning from 24 to 120 h post-irradiation, to monitor spheroid growth dynamics. Data are presented as mean ± standard deviation, with fitted trend lines and associated 95% confidence intervals. Graphical representation was generated using BioRender.com. (J) Representative brightfield images of control and 6 Gy CIRT-irradiated T98G spheroids acquired at baseline (0 h) and 24-h intervals up to 120 h. Scale bar is 500 µm. (K) Spheroid diameter growth rates, expressed as linear regression slopes, assessed for non-irradiated and irradiated groups (photons, protons and carbon ions) in T98G, Saos-2 and U2-OS cell lines. (L) RBE values derived from proton and carbon ion exposure in the three 3D tumour spheroids.

In T98G spheroids, exposure to photon irradiation resulted in progressively more negative slope values with increasing dose, measured at −0.225, −0.3226 and −0.3913 for 2, 4 and 6 Gy, respectively. A more marked reduction in spheroid growth was observed following proton irradiation, with slopes ranging from −0.2579 at 2 Gy to −0.4940 at 6 Gy. Carbon ion irradiation induced the most pronounced decrease, with slopes of −0.2952, −0.4623 and −0.5881 corresponding to 2, 4 and 6 Gy. Saos-2 spheroids showed a marked reduction in growth rate under photon irradiation, with slope values of −0.3651 at 2 Gy and −0.7262 at 4 Gy, followed by a less negative value at 6 Gy (−0.1095). Under proton irradiation, slope values were less pronounced, ranging from −0.0496 at 2 Gy to −0.1079 at 6 Gy. Similarly, carbon ion irradiation resulted in slopes between −0.0519 and −0.1492, with a marked decrease at 4 Gy (−0.8095). In U2-OS spheroids, photon irradiation had minimal effect on growth rate, with slope values remaining close to zero across all doses (−0.0059 to −0.0085). Slightly more negative slopes were observed following proton exposure (−0.0289 to −0.0972) and carbon ion irradiation (−0.0190 to −0.1488), indicating a more gradual but consistent reduction in spheroid growth (Figure 4K) [22].

Irradiation effects on 3D cell cultures were quantified by calculating spheroid growth rates from diameter measurements recorded every 24 h up to 120 h post-irradiation. Growth rates were normalised to controls to assess inhibition and used to calculate the RBE of proton and carbon ion RT relative to conventional X-rays at equivalent endpoints. Our findings indicate that carbon ions consistently exert enhanced biological efficacy, with RBE values markedly higher than those observed in 2D cultures. The glioblastoma cell line T98G exhibited the highest RBE, quantified at 4.0, compared to an RBE of 1.0 for protons. Within the osteosarcoma models, U2-OS and Saos-2 presented carbon ion RBE values of 3.8 and 3.6, respectively, whereas proton RBE values for both cell lines remained approximately 1.0 (Figure 4L).

Spheroid viability in T98G, Saos-2 and U2-OS cells was assessed 5 days post-irradiation using PrestoBlue (Figure 5A–C). In T98G spheroids, viability at 2 Gy was 91.8 ± 2.2% (photons), 89.0 ± 1.6% (protons) and 86.0 ± 3.9% (carbon ions), decreasing to 84.8 ± 5.5%, 83.0 ± 4.2% and 79.8 ± 6.3% at 6 Gy. U2-OS spheroids showed similar trends, with 2 Gy viabilities of 92.4 ± 2.4%, 89.6 ± 4.5% and 86.0 ± 5.7%, dropping to 86.0 ± 5.7%, 85.0 ± 3.9% and 82.0 ± 5.7% at 6 Gy. Saos-2 spheroids were most radiosensitive, with viabilities of 92.8 ± 5.1%, 88.0 ± 2.1% and 82.6 ± 4.3% at 2 Gy, falling to 85.2 ± 3.7%, 80.4 ± 6.3% and 73.8 ± 6.3% at 6 Gy.

Spheroid invasion was quantitatively assessed in T98G, Saos-2 and U2-OS cell lines after exposure to photons, protons and carbon ions at doses of 2, 4 and 6 Gy over 120 h (Figure 5G). Control spheroids displayed a steady increase in invasion over time, with T98G controls reaching 2.49 ± 0.18 and U2-OS controls 1.85 ± 0.15 by 120 h.

**Figure 4b:**
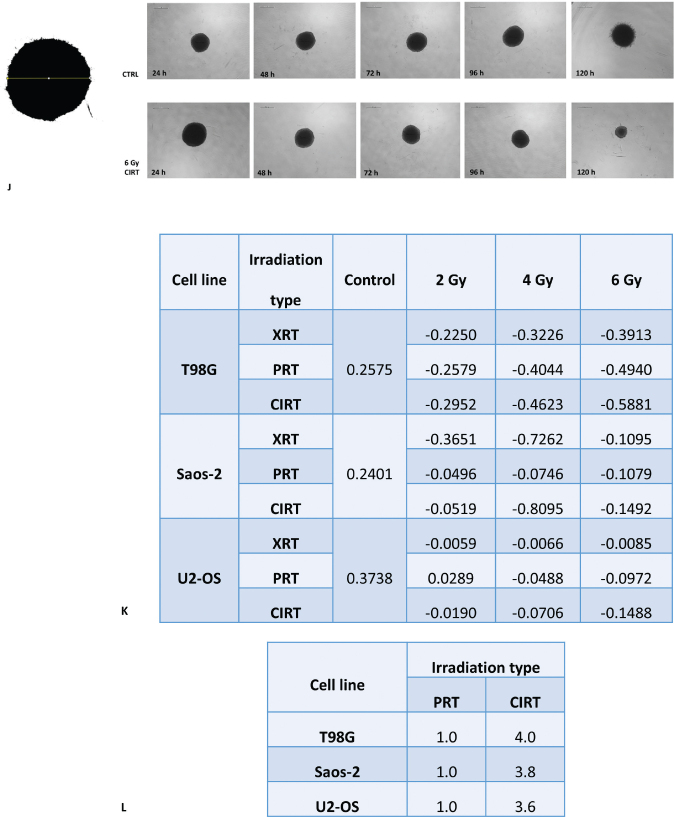
Growth curves of 3D spheroids derived from T98G (A–C), Saos-2 (D–F) and U2-OS (G–I) cell lines, comparing non-irradiated controls to spheroids exposed to photon, proton and carbon ion irradiation. Baseline measurements (time point 0) correspond to spheroid size before irradiation. Subsequent measurements were performed at 24-h intervals, spanning from 24 to 120 h post-irradiation, to monitor spheroid growth dynamics. Data are presented as mean ± standard deviation, with fitted trend lines and associated 95% confidence intervals. Graphical representation was generated using BioRender.com. (J) Representative brightfield images of control and 6 Gy CIRT-irradiated T98G spheroids acquired at baseline (0 h) and 24-h intervals up to 120 h. Scale bar is 500 µm. (K) Spheroid diameter growth rates, expressed as linear regression slopes, assessed for non-irradiated and irradiated groups (photons, protons and carbon ions) in T98G, Saos-2 and U2-OS cell lines. (L) RBE values derived from proton and carbon ion exposure in the three 3D tumour spheroids.

**Figure 5:**
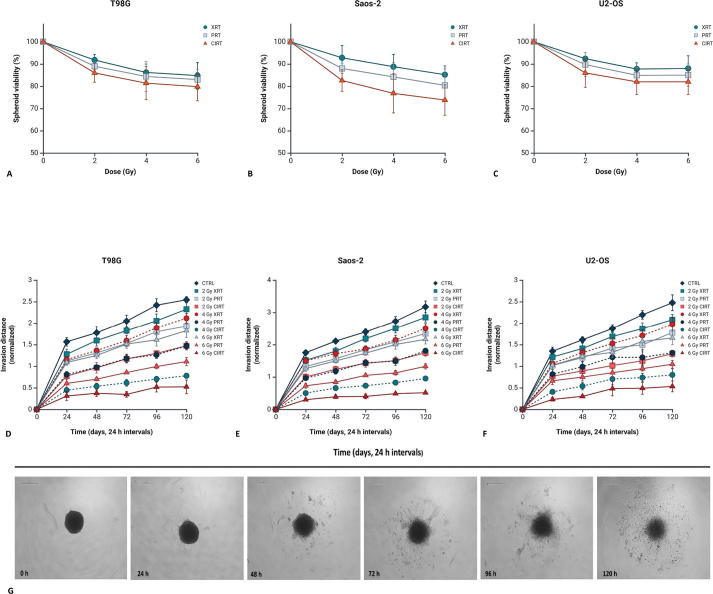
Spheroid viability evaluated 5 days following irradiation with photons, protons or carbon ions and expressed as normalised percentages for (A) T98G, (B) Saos-2 and (C) U2-OS cell lines. Data represent the mean ± standard deviation from three independent experiments. Spheroid invasion evaluated 5 days following exposure to XRT, PRT or CIRT for (D) T98G, (E) Saos-2 and (F) U2-OS cells. Results are expressed as mean ± SD from three independent experiments. To enhance clarity and highlight treatment-specific effects, the y-axis scale was individually optimised for each graph. Figures created using BioRender.com. (G) Representative brightfield images of control T98G spheroids captured at 24-h intervals up to 120 h, with 0 h representing the baseline. Scale bar is 500 µm.

In T98G spheroids, photons caused a modest decrease in invasion at 24 h post-irradiation, with values of 1.12 ± 0.06 compared to 1.38 ± 0.09 in controls. Proton irradiation led to a stronger suppression, yielding 0.68 ± 0.07 at 6 Gy, while carbon ion irradiation demonstrated the most pronounced inhibition, reducing invasion to 0.23 ± 0.03 (6 Gy) at 24 h. This suppression persisted throughout the time course; at 120 h, carbon ion-treated T98G spheroids at 6 Gy showed invasion values of 0.52 ± 0.12 compared to controls. Similarly, Saos-2 and U2-OS spheroids exposed to carbon ion irradiation demonstrated marked invasion inhibition relative to photons and protons. For instance, at 48 h post 4 Gy exposure, Saos-2 spheroids showed invasion values of 0.47 ± 0.06 compared to 1.75 ± 0.12 in controls. U2OS spheroids followed a similar pattern, with invasion indices of 0.50 ± 0.08 after 4 Gy carbon ion irradiation at 48 h, versus 1.70 ± 0.15 in untreated controls. Additionally, U2-OS spheroids exhibited significant invasion suppression following carbon ion irradiation. At 48 h, control invasion was 1.76 ± 0.11, while carbon ion treatment at 4 Gy reduced invasion to 0.75 ± 0.08. At 72 h and 6 Gy, invasion remained suppressed in U2OS spheroids exposed to carbon ions, with values of 0.53 ± 0.14 compared to 2.02 ± 0.16 in controls. Photon and proton irradiations resulted in intermediate reductions, with proton-treated spheroids generally showing lower invasion than photons but higher than carbon ions (Figure 5D–F).

**Figure 6:**
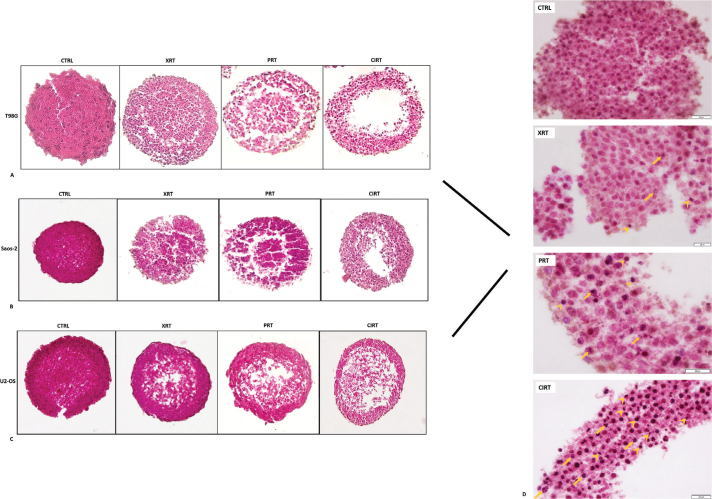
Histological sections of (A) T98G, (B) Saos-2 and (C) U2-OS spheroids stained with haematoxylin-eosin, following irradiation. Spheroids were fixed and sectioned 7 days after exposure to 6 Gy of photons, protons or carbon ions. Representative images illustrate morphological changes in response to different radiation qualities across the three tumour cell lines. Scale bar is 50 µm. (D) Representative H&E-stained sections of spheroid fragments under control conditions and following irradiation with photons, protons and carbon ions (top to bottom). Arrows indicate fragmented nuclei, whereas arrowheads denote pyknotic ones. Images were acquired at 100X magnification, and scale bar is 20 µm.

Histological analysis of H&E-stained spheroid sections at 7 days following exposure to 6 Gy of irradiation revealed distinct morphological alterations depending on the radiation modality (Figure 6). In non-irradiated control spheroids, a well-preserved and organised architecture was observed. Cells displayed uniform morphology with even spatial distribution throughout the spheroid. Nuclei appeared round, with minimal heterochromatin condensation, and cytoplasmic regions exhibited characteristic eosinophilic staining, consistent with intact and viable cellular structures. In contrast, spheroids exposed to photons demonstrated substantial architectural disruption. A marked decrease in cell density was evident, particularly at the periphery, accompanied by widespread regions of cellular degeneration. These areas contained numerous pyknotic and fragmented nuclei, indicating extensive nuclear damage and compromised cell viability. Proton irradiation induced prominent cytoplasmic degeneration, particularly within the spheroid core. Pyknotic and fragmented nuclei were frequently observed, and degenerative changes extended towards the spheroid surface, reflecting diffuse structural deterioration. Exposure to carbon ions resulted in the most pronounced morphological damage. Spheroids exhibited extensive structural disintegration, with widespread degeneration throughout the entire mass and a high prevalence of damaged and dying cells. Interestingly, the characteristic hypoxic core evident in control spheroids was absent, indicating widespread structural disintegration and loss of spheroid organisation. In Saos-2 spheroids, 2 Gy XRT resulted in a moderate increase in invasion (1.11 ± 0.07), which became more pronounced at 4 Gy (1.23 ± 0.09), and peaked at 6 Gy (1.36 ± 0.12), suggesting a potential radiation-induced stimulation of invasive behaviour. Proton irradiation led to a different trend: at 2 Gy, invasion remained comparable to control (1.02 ± 0.08), while 4 Gy showed a modest increase (1.10 ± 0.06), and 6 Gy reached 1.15 ± 0.05, indicating only a mild enhancement. Conversely, carbon-ion exposure induced a marked anti-invasive effect across all doses. At 2 Gy CIRT, Saos-2 invasion was reduced to 0.87 ± 0.07, further declining to 0.72 ± 0.05 at 4 Gy and reaching a significant suppression at 6 Gy (0.58 ± 0.09).

## Discussion and conclusion

This study evaluated the radiobiological effects of photon, proton and carbon ion irradiation on T98G, Saos-2 and U2-OS cells cultured as 2D monolayers and 3D spheroids. Radioresistant cell lines were selected to rigorously assess PT (only abbreviation) in a challenging context and address the limitations of 2D models. CIRT (only abbreviation) consistently showed superior efficacy over protons and photons in reducing survival, viability and invasion across all cell lines and models, while protons were intermediate and photons least effective.

An increase in invasion was observed following proton irradiation in 2D assays, with a dose-dependent trend. Although counterintuitive, similar effects have been reported after low-LET irradiation and are generally attributed to stress-mediated adaptive responses in surviving cells rather than enhanced tumour aggressiveness [40]. Mechanistically, low-LET radiation can activate signalling pathways involved in cytoskeletal remodelling and cell motility, including Rho GTPase-dependent regulation of actin dynamics, focal adhesion turnover and integrin-mediated cell–matrix interactions. Radiation-induced upregulation of matrix metalloproteinases and transient epithelial-mesenchymal transition-like features has also been reported, facilitating invasion through extracellular matrices [41–42]. Because low-LET irradiation predominantly induces repairable DNA damage, increasing dose may amplify these signalling responses while preserving cellular motility [28, 29].

Notably, 3D spheroids exhibited higher apparent radioresistance than 2D cultures, reflecting microenvironmental complexity, including cell–cell and cell–matrix interactions, nutrient and oxygen gradients and spatial heterogeneity [11, 30–34]. High-LET carbon ions partially overcame these factors, showing consistent effects across 2D and 3D systems [33]. Spheroid models also enabled more physiologically relevant invasion assays, capturing dynamic, spatially organised behaviour that better approximates *in vivo* tumours [35–43].

Cell line-specific differences were evident, since T98G glioblastoma displayed pronounced radioresistance, while osteosarcoma lines maintained significant resistance in 3D. Limitations include the absence of stromal or immune components, static spheroid conditions and a short 5-day timeframe for RBE assessment, which may not fully capture long-term effects [39].

Despite these constraints, this is the first systematic study comparing multiple radioresistant lines under 2D and 3D conditions across low- and high-LET irradiation. Our findings highlight the utility of 3D spheroids as a more clinically relevant preclinical platform for evaluating PT and informing future *in vivo* studies.

To our knowledge, this study is the first to quantitatively estimate RBE using tumour spheroids, establishing a novel framework for assessing radiation quality in physiologically relevant 3D models. We show that photons, protons and carbon ions differently affect survival, viability and invasion of intrinsically radioresistant cell lines, with carbon ions most effectively reducing cell viability, metastatic behaviour and spheroid integrity. 3D spheroids captured key structural and functional features of *in vivo* tumours, offering greater physiological relevance than 2D cultures. The consistent efficacy of carbon ions across both models underscores their therapeutic potential, while differences between 2D and 3D responses highlight the limitations of monolayer assays and support the use of advanced *in vitro* systems for more reliable preclinical evaluation of emerging radiation modalities.

## Supplementary Material



## Data Availability

Research data are stored in an institutional repository and will be shared upon reasonable request to the corresponding author.
